# The Teacher, the Physician and the Person: Exploring Causal Connections between Teaching Performance and Role Model Types Using Directed Acyclic Graphs

**DOI:** 10.1371/journal.pone.0069449

**Published:** 2013-07-23

**Authors:** Benjamin C. M. Boerebach, Kiki M. J. M. H. Lombarts, Albert J. J. Scherpbier, Onyebuchi A. Arah

**Affiliations:** 1 Professional Performance Research Group, Center for Evidence-Based Education, Academic Medical Center, University of Amsterdam, Amsterdam, The Netherlands; 2 Faculty of Health, Medicine and Life Sciences, Maastricht University, Maastricht, The Netherlands; 3 Department of Epidemiology, Fielding School of Public Health, University of California Los Angeles, Los Angeles, California, United States of America; 4 University of California Los Angeles, Center for Health Policy Research, Los Angeles, California, United States of America; Sapienza University of Rome, Italy

## Abstract

**Background:**

In fledgling areas of research, evidence supporting causal assumptions is often scarce due to the small number of empirical studies conducted. In many studies it remains unclear what impact explicit and implicit causal assumptions have on the research findings; only the primary assumptions of the researchers are often presented. This is particularly true for research on the effect of faculty’s teaching performance on their role modeling. Therefore, there is a need for robust frameworks and methods for transparent formal presentation of the underlying causal assumptions used in assessing the causal effects of teaching performance on role modeling. This study explores the effects of different (plausible) causal assumptions on research outcomes.

**Methods:**

This study revisits a previously published study about the influence of faculty’s teaching performance on their role modeling (as teacher-supervisor, physician and person). We drew eight directed acyclic graphs (DAGs) to visually represent different plausible causal relationships between the variables under study. These DAGs were subsequently translated into corresponding statistical models, and regression analyses were performed to estimate the associations between teaching performance and role modeling.

**Results:**

The different causal models were compatible with major differences in the magnitude of the relationship between faculty’s teaching performance and their role modeling. Odds ratios for the associations between teaching performance and the three role model types ranged from 31.1 to 73.6 for the teacher-supervisor role, from 3.7 to 15.5 for the physician role, and from 2.8 to 13.8 for the person role.

**Conclusions:**

Different sets of assumptions about causal relationships in role modeling research can be visually depicted using DAGs, which are then used to guide both statistical analysis and interpretation of results. Since study conclusions can be sensitive to different causal assumptions, results should be interpreted in the light of causal assumptions made in each study.

## Introduction

Role modeling research is a relatively new area in the emerging field of medical education research. Several studies have explored the attributes of good role models. However, empirical studies on the impact of faculty’s behaviors and attributes on their role modeling are limited. [1] As with other budding disciplines, cause and effect are therefore not yet supported by many empirical studies. When designing and analyzing a study on relationships between exposures and outcomes, there is often a need to make certain causal assumptions. Indeed, it can be argued that many investigative studies in medical education implicitly appeal to causal assumptions and interpretations. For instance, researchers who use quantitative methods like regression models and structural equation models need to make causal assumptions in their analyses. These assumptions are usually made early on a study. [2] However, the implications of causal assumptions on research findings often remain unclear and unexplored.

The aim of this study is to illustrate how using different causal assumptions can impact research findings in role modeling research. To this end, we build on our recently published study regarding the impact of faculty’s teaching performance on them being seen as role models. [3] Since systematic exploration of the effects of causal assumptions is new to the field of medical education research, this paper can serve as a scholarly example on how researchers can examine causal relationships between variables in their studies. We begin by briefly describing the previously investigated relationship between teaching performance and role modeling. Next we introduce the now well-established graphical tools *directed acyclic graphs* (DAGs) that are new to the field of medical education research. [4]–[6] Then we present our theoretical assumptions about the (causal) connections between teaching performance and role modeling. Finally we translate the DAGs into statistical models, perform the statistical analyses, and compare and interpret the results from the different models vis-à-vis our causal assumptions.

### Role Modeling and Teaching Performance

Role modeling is a relatively new and hot topic in medical education research. [7] Role modeling is considered a teaching strategy since medical students and residents learn by observation of faculty. [7], [8] It has been suggested that students and residents distinguish between a three role model typology: they may see faculty as a role model *teacher-supervisor*, *physician,* and *person*. [9], [10] In many modern postgraduate medical education settings, residents can learn from a group of faculty or supervisors, and thus are not tied to one specific faculty for a long period. Consequently, residents learn different competencies from multiple faculty who fulfill distinct functions as role models. A recent study revealed that residents actually search for and distinguish between different role models for the roles of teacher, physician and person. [3] A few descriptive studies reported characteristics of faculty that might enhance their role modeling in these different capacities. [9]–[13] These characteristics include teaching qualities, clinical qualities, and personal qualities. Empirical evidence supporting the influence of these qualities on the different role model types is still scarce. In a recently published empirical study, [3] we explored the relationship between faculty’s teaching performance and their role modeling. Results of that study suggest that faculty’s teaching performance could impact their role modeling as teacher-supervisor, physician, and person. Although faculty’s teaching performance was more prone to influence their role modeling as teacher-supervisor, teaching performance was also found to be highly associated with the physician and person role model types.

Our previous study included multiple analyses, to provide a detailed overview of the impact of faculty’s teaching performance on their role modeling in different roles as teacher-supervisor, physician and person. In that study, we had to make assumptions about the causal relationship between teaching performance and role modeling. We assumed that faculty who enhanced their teaching performance were more likely to be seen as better role models. This relationship was supported by the scarce literature available on role modeling. [7] Furthermore, we had to specify if there were any causal relationships between the three role model types; for example, did role modeling as a person enhance role modeling as a teacher-supervisor or physician? Although we tried to find support for these assumptions in the limited literature available on role modeling, [1], [13], [14] the absence of a clear theoretical framework allowed us to make different assumptions. Given previous studies, we assumed that the three role model types were not causally interrelated. In this study, we further explore the different causal models not explored in our previous study, mainly illustratively.

The specific research questions explored in our current study were: 1) what are the potential causal relationships between teaching performance and the three role model types; and 2) how do these different causal assumptions impact the associations between teaching performance and the role model types? We explored the main plausible causal models on this topic, to gain insights into the influence of faculty’s teaching performance on their role modeling as teacher-supervisor, physician, and person. In the absence of a clear and generally accepted theoretical framework supported by empirical evidence, it was not our aim to search for one “best model” or to compare the models in terms of statistical goodness-of-fit.

## Methods

Waiver of ethical approval was provided by the Institutional Review Board of the Academic Medical Center of the University of Amsterdam, Amsterdam, The Netherlands. A waiver was provided because ethical approval for this study was not required under Dutch law.

### Causal Diagrams

In epidemiology, computer science, social sciences and other quantitative disciplines, graphical models embodied by directed acyclic graphs (DAGs) are increasingly used to illustrate causal relationships between variables. [6], [15], [16] DAGs have a long history that can be traced back to path diagrams that graphically represent structural equations models (SEM) often used by medical education researchers and social scientists. Over the last two decades, graphical models were generalized and extended to allow for nonparametric, probabilistic, causal, and functional interpretations beyond their more common parametric (linear) purview as path diagrams. Backed by a set of elegant mathematical machinery, often embodied by the graphs, DAGs tend to be user-friendly for both technical and non-technical researchers. DAGs provide researchers with a useful tool for visualizing their research question(s), conveying assumptions in a transparent manner, deciding on a sufficient set of confounders to include in their analysis for effect estimation, and recognizing when to take more measurements before proceeding further. Because DAGs transcend statistical methods, they can be used in any situation where causal relationships between variables on a specific topic need to be visualized. While path analysis or SEM accompanied by path diagrams also use graphical models, they require specific statistical assumptions about linearity and multivariate normality, while DAGs can be used regardless of the subsequent statistical assumptions made. That is, DAGs are non-parametric representations of causal models while path diagrams are the parametric representations of SEM. Furthermore, one of the most important developments in modern causality is the distinction between causal concepts and statistical concepts. [6] Statistical concepts are determined by the probability distributions of the variables under study and can provide information about associations between such variables. However, causal relations cannot be determined by probability distributions alone. Statistical tools can show if there is an association between two variables, but they are unable to determine if those two variables are associated by a direct causal effect, an indirect causal effect, a (unmeasured) common cause of the two variables, conditioning on a common consequence of the two variables, or a combination of the above described possibilities. Based on background knowledge, real world observations and experiments, researchers can construct appropriate DAGs for depicting causal relations in a specific study. Subsequently, researchers can use the DAGs to guide their choice of covariates for confounding control with respect to target causal effects, and then choose appropriate statistical techniques for the analysis while taking into account their data and the assumptions required for the chosen statistical techniques. Since DAGs are new to medical education research, we will first briefly introduce the most commonly used features of DAGs. In this description of DAGs, we will use teaching performance and the three role model types in order to illustrate our points.

**Figure 1:**
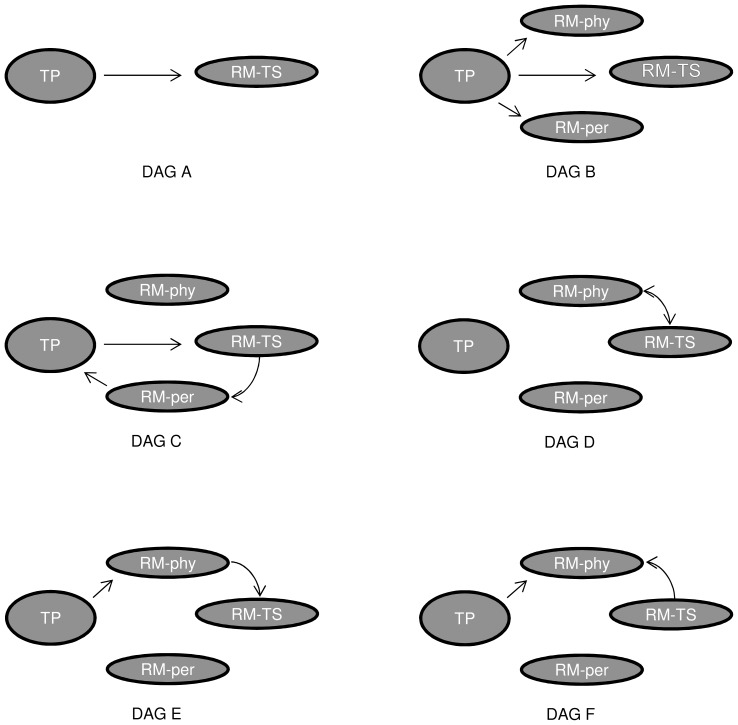
Examples of directed acyclic graphs (DAGs) relating teaching performance and role model types. DAG A: TP causes RM-TS (a direct path). DAG B: TP causes the three types of role modeling (that is, the three role modeling types share a common cause, TP). DAG C: an ineligible cyclic path. DAG D: a bidirectional path. DAG E: RM-phy mediates the path from TP to RM-TS. DAG F: RM-phy is a collider variable between TP and RM-TS.

An example of a DAG is illustrated in figure 1 DAG A, where teaching performance (TP) is the predictor or exposure variable and the role model type teacher-supervisor (RM-TS) is the outcome. A DAG can be expanded by adding more variables. In figure 1 DAG B, the DAG is expanded by adding two extra outcome variables, namely role model type as physician (RM-phy) and as person (RM-per). In theory, a DAG can have a large number of variables, but for practical reasons it is recommended to limit the number of variables to those that are most important for answering the research question [6].

In a graph, variables represent nodes or vertices. Variables are connected by arcs or edges. Adjacent variables are those connected by an edge, while adjacent edges are those that meet at a variable. An edge is usually an arrow where the variable at the tail of the arrow is called a parent while the variable at the arrowhead is called a child. An arrow represents a “direct” causal effect, often called as such because intermediate variables have been omitted. A sequence of adjacent edges or arrows is called a *path*. A directed path is one formed by following arrows aligned only from their tails to heads. A directed path is causal. An example of a *directed path* is found in figure 1 DAG A, where the path between TP and RM-TS is a directed path towards RM-TS. The causal relationship is simple: TP causes RM-TS. An acyclic graph is one without a feedback loop meaning no variable causes itself. Therefore, a directed acyclic graph (DAG) is a graph with tail-to-head arrows as edges and no feedback loops (an ineligible feedback loop is illustrated in figure 1 DAG C). Often, a (dashed) bidirectional arrow is used to depict an omitted common cause or parent. An example of a *bidirectional path* is the path between RM-TS and RM-phy in figure 1 DAG D.

A *mediating* variable is one that intercepts the causal pathway between two variables. For example, RM-phy is a *mediator* (TP→RM-phy→RM-TS) in figure 1 DAG E. A *collider* is a variable in a path with at least two arrows pointing into it. A collider blocks a path between two other variables. An example of a collider variable is RM-phy in figure 1 DAG F on the path from TP to RM-TS through C (TP→RM-phy←RM-TS).

An open undirected path between any two variables in a DAG is called a biasing path. In an unconditional DAG, all biasing paths are backdoor paths. A backdoor path is a biasing path that begins with an arrow pointing into the exposure (say, TP) and ends with an arrow pointing into the outcome (say, RM-TS). The simplest example would be a path formed by drawing a common cause of both TP and RM-TS (say, U) into DAG A of Figure 1. One of the most important results of graph theory and DAGs is the backdoor criterion which instructs us to find a sufficient set of variables to block the open biasing path or backdoor. [5], [6] Variable selection for control of confounding or to close backdoors is central to identification and estimation of causal effects. It is, perhaps, not surprising that variable selection has spurred several misconceptions in the literature [6], [17].

### DAGs for Teaching Performance and Role Modeling

**Figure 2:**
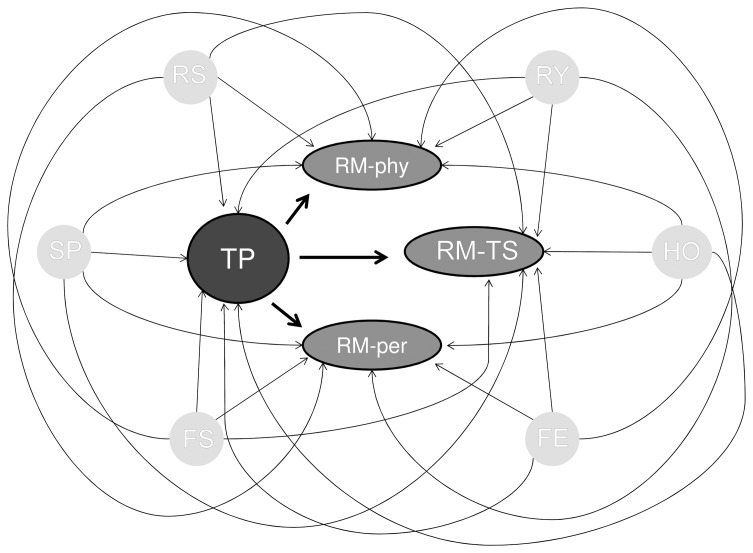
DAG of the relationship between teaching performance and role modeling. TP = teaching performance; RM-phy = role model physician; RM-TS = role model teacher-supervisor; RM-per = role model person; RS = residents’ sex; RY = residents’ residency year; FS = faculty’s sex; FE = faculty’s experience; HO = hospital; SP = specialty.

**Figure 3:**
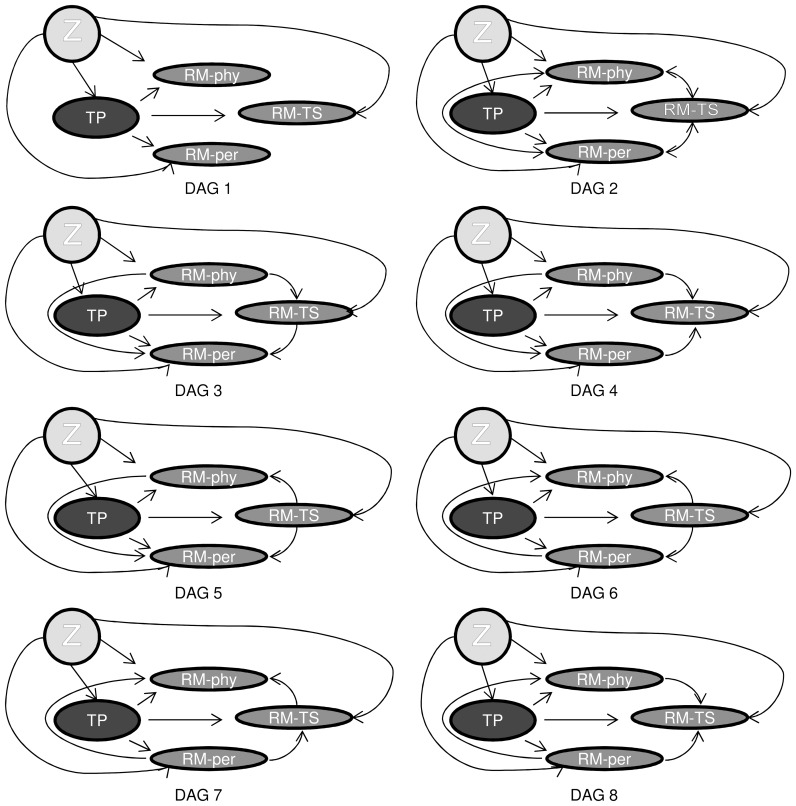
DAGs of the different causal relationships between teaching performance and the different role model types. TP = teaching performance; RM-phy = role model physician; RM-TS = role model teacher-supervisor; RM-per = role model person; Z depicts the covariates namely, faculty’s sex and years of experience, residents’ sex and residency training year, hospital and specialty. (This collapsing of the covariates into one variable Z was only intended to make the DAGs more legible in this illustrative study; but we discourage doing so in actual applications.).

Next, we drew all plausible causal relationships between the variables teaching performance and the role model types, using the DAGs introduced above. We included the main study variables teaching performance (TP) and the three role model types, teacher-supervisor (RM-TS), physician (RM-phy), and person (RM-per) in all DAGs. In addition, we included the covariates faculty’s sex (FS) and experience (FE), residents’ sex (RS) and residency year (RY), hospital (HO), and specialty (SP) in the DAGs. The relationship between these covariates, the predictor variable (TP), and the outcome variables (RM-TS, RM-phy and RM-per) were fixed in all DAGs (i.e. each of these covariates impacted both the exposure TP and the outcome role model types in all models; thus they were identified as confounders for the relationship between TP and the role model types). [3], [13] Because there was some evidence that supported a causal relationship from teaching performance towards the different role model types (and not reverse), [9]–[12] these relationships were also fixed in all DAGs. The DAG in Figure 2 shows all causal relationships among the variables that were fixed in subsequent DAGs. To make the subsequent DAGs simpler to read, all covariates were visualized as one variable Z (as in figure 3, DAG 1). The interconnectivity between the role model types could then be defined. To our knowledge there was no scientific evidence about the causal relationships between the three role model types. Therefore we drew all possible (combinations of) paths between the role model types that we considered plausible. In the first DAG there were no causal relationships between the role model types (Figure 3, DAG 1). In the second DAG, the role model types had no direct causal effect on each other, but they shared connections with other variables outside this graph (Figure 3, DAG 2). In the other DAGs, causal relationships between the role model types were possible, such that high performance on one role model type could have caused enhanced performance on another role model type. In these DAGs (Figure 3, DAG 3-DAG 8), one role model type could *mediate* the relationship between teaching performance and another role model type.

### From DAGs to Statistical Models for Estimation

Next, the target causal relationships depicted in the DAGs were translated into statistical models. Since we were interested in the influence of a predictor variable (teaching performance) on certain outcome variables (the three role model types), we used regression models to estimate the “effects” under the (untestable) assumption of ‘no uncontrolled confounding’ as well as assumptions of no bias due to measurement error and selection bias. All regression models included teaching performance as the exposure (or main predictor) variable and one of the three role model types as the outcome variable. In some models, adjustment for role model types other than the outcome role model type was required, such as when non-mediated effects were under consideration and the other role model types could serve as mediators of the relationship between teaching performance and the outcome role model type. We explain how we used the DAGs to guide our analysis in the current study below.

**Figure 4:**
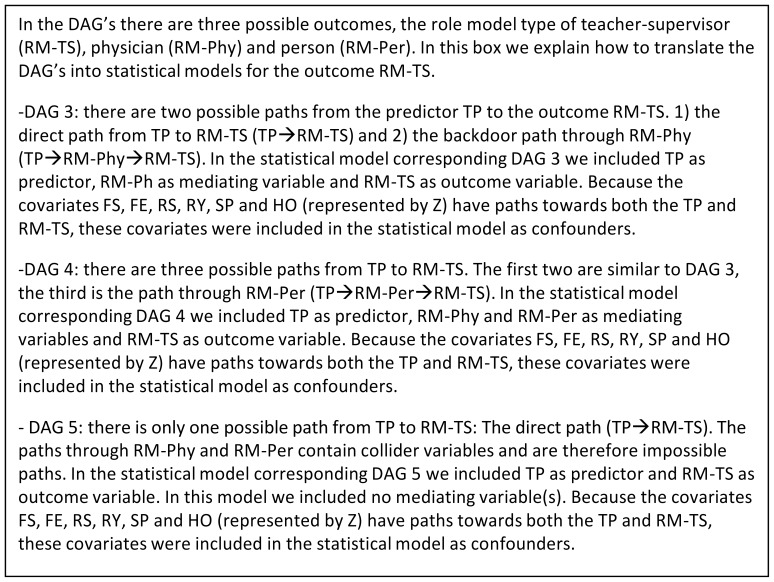
DAGs and models.

Because the role model types in DAG 1 of Figure 3 were not causally related (there was no path between the role model types), the regression models that correspond to DAG 1 were not adjusted for mediating variables. By DAG rules, the relationship between TP and RM-TS in DAG 2 was not confounded by either RM-phys or RM-per and could, therefore, be estimated without conditioning on either. Therefore, the result for DAG 2 was comparable to that of DAG 1. The circumstances for DAGs 3 to DAG 8 were more complicated. In Figure 4, we elaborate on an example of how we used the DAGs to guide the specifications of corresponding regression models.

Ultimately, we ran four unique models for each of the three role model types as an outcome measure (resulting in a total of twelve models). For each role model type outcome, the first model only included teaching performance as the main predictor and included no mediating variables. The second model included teaching performance as the main predictor and one of the two remaining role model types as a mediating variable. The third model included teaching performance as the main predictor and the other remaining role model type (i.e. the one not used in model 2) as a mediating variable. The fourth model included the teaching performance as the main predictor and both remaining role model types as mediating variables. Because the covariates faculty’s sex and years of experience, residents’ sex and residency training year, hospital and specialty had arrows pointing towards both the exposure variable teaching performance and the outcome role model types in all DAGs, they were identified as confounders of the relationship between teaching performance and role modeling. Therefore the variables were included as confounders in all regression models.

### Data on Teaching Performance and Role Modeling

The data on faculty’s teaching performance and the role model types were obtained in our previous study. [3] In that study, we used a validated web-based system called System for Evaluation of Teaching Qualities (SETQ),[18]–[21] to obtain data on faculty’s teaching performance and their role modeling. The SETQ questionnaires used 21 core items to evaluate faculty’s teaching performance and three additional items to evaluate their role modeling. The SETQ core items were all preceded by the statement “During my residency training my attending faculty generally…”. Examples of the items were: … stimulates residents to bring up problems; … listens attentively to residents; and … offers suggestions for improvement. The role model items were quoted as: “During my residency, this faculty is a role model to me in his/her role as… i) teacher/supervisor; ii) physician; iii) a person”. All items were scored on 5-point Likert scale: 1 = “strongly disagree”, 2 = “disagree”, 3 = “neutral”, 4 = “agree”, and 5 = “strongly agree”, and there was an additional option “I cannot judge”. Previous validation studies of the SETQ instruments included, but were not limited to, exploratory factor analyses, internal consistency analyses, inter-scale correlations, scale versus global rating correlations, and item versus total scale correlations. These validation studies showed that the SETQ instruments were reliable and valid for measuring faculty’s teaching performance in various settings. [18]–[21] Those previous SETQ validation studies suggested a five-factor structure for teaching performance: *learning climate, professional attitude towards residents, communication of goals, evaluation of residents* and *feedback*. A Principal component analysis with varimax rotation of our current study data yielded the same five-factor structure. Additionally, internal consistency analysis yielded Cronbach’s alphas ranging from 0.89 to 0.92 for the five factors and 0.96 for teaching performance overall (i.e., all items combined). The full results of the psychometric analyses are available in Appendix S1.

The *teaching performance* variable used in the current study was an average score of the 21 core items from the SETQ questionnaires. The outcome variables used in this study were residents’ perceptions of faculty’s role modelling on the three different *role models types*. Participants who provided the data for the study were 219 residents, who evaluated 423 faculty. Faculty and residents worked in the anesthesiology, internal medicine, obstetrics & gynecology, pediatrics or surgery departments of eleven different teaching hospitals in The Netherlands. In total, residents completed 2111 evaluations, yielding, on average, five resident evaluations per faculty. For more information on the setting and background characteristics of study participants we refer the reader to our previous study [3].

### Statistical Analysis

Data were analyzed using the regression models described above. We began by checking the statistical assumptions required for performing parametric analyses. [2] Since these statistical assumptions were met, we proceeded to choose an appropriate parametric model for estimating the associations between faculty’s teaching performance and their role modeling. We used generalized estimating equations (GEE) to adjust for clustering on hospital, specialty, faculty, and resident level. [2] We used ordinal logistic GEE models, as our data contained ordinal outcome variables. The associations between faculty’s teaching performance and their role modeling were presented as odds ratios and 95% confidence intervals. Data were analyzed using IBM SPSS Statistics 21 for Windows operating system.

## Results

In all models (table 1), teaching performance was positively associated with the role model types of teacher-supervisor, physician, and person. Overall, the magnitudes of the associations were higher for the teacher-supervisor role model (RM-TS) compared to the other two role model types. In the models that included other role model types as mediating variables, the magnitude of the effect of teaching performance on the outcome was reduced. In all models, variables that were included as mediators had a substantial associations with the outcome.

**Table 1:** Associations between teaching performance and the role model types for the different DAGs.

Outcome model	Corresponding DAG number	Exposure(s)	Odds Ratio (95% Confidence Interval)
Outcome: Role model as a teacher-supervisor			
RM-TS1	1, 2, 5, 6	Teaching performance	71.55 (53.73–95.27)
RM-TS2	3	Teaching performance	41.95 (31.01–56.73)
		Role model physician	2.41 (2.00–.92)
RM-TS3	7	Teaching performance	39.10 (28.78–53.12)
		Role model person	2.44 (2.07–2.87)
RM-TS4	4, 8	Teaching performance	31.06 (22.70–42.50)
		Role model physician	1.84 (1.50–2.25)
		Role model person	2.02 (1.70–2.40)
Outcome: Role model as a physician			
RM-phy1	1, 2, 3, 4	Teaching performance	15.82 (12.62–19.82)
RM-phy2	5	Teaching performance	5.52 (4.14–7.37)
		Role model teacher-supervisor	2.79 (2.28–3.40)
RM-phy3	8	Teaching performance	6.61 (5.14–8.51)
		Role model person	3.31 (2.78–3.95)
RM-phy4	6, 7	Teaching performance	3.70 (2.75–4.99)
		Role model teacher-supervisor	2.00 (1.62–2.46)
		Role model person	2.81 (2.35–3.38)
Outcome: Role model as a person			
RM-per1	1, 2, 7, 8	Teaching performance	13.65 (11.17–16.70)
RM-per2	6	Teaching performance	4.56 (3.50–5.95)
		Role model teacher-supervisor	2.96 (2.45–3.59)
RM-per3	4	Teaching performance	6.14 (4.89–7.72)
		Role model physician	3.61 (3.00–4.35)
RM-per4	3, 5	Teaching performance	2.80 (2.12–3.69)
		Role model teacher-supervisor	2.41 (1.98–2.93)
		Role model physician	3.15 (2.61–3.81)

All models were additionally adjusted for these covariates: faculty’s sex and years of experience, residents’ sex and residency training year, hospital and specialty.

For models with the role model type of teacher-supervisor as the outcome, the odds ratio (95% confidence interval) was 73.6 (54.8–98.8) for the model that not included any mediating variables (RM-TS1), 41.9 (31.0–56.7) for the model that included the role model physician as a mediating variable (RM-TS2), 39.1 (28.8–53.1) for the model that included the role model person as a mediating variable (RM-TS3), and 31.1 (22.7–42.5) for the model that included both role model types as mediating variables (RM-TS4).

For the models with the role model type of physician as outcome measure, the odds ratios ranged from 15.5 (12.3–19.5) for model RM-phy1 to 3.70 (2.75–4.99) for model RM-phy4 (table 1). For the models with the role model person as the outcome measure, the odds ratios ranged from 13.8 (11.2–17.0) for model RM-per1 to 2.8 (2.12–3.69) for model RM-per4 (table 1).

## Discussion

The aim of this study was to explore the impact of applying different causal assumptions regarding the interrelationships of role model types and teaching performance. Applying different causal models resulted in large differences in the associations between faculty’s teaching performance and their role modeling. To our knowledge, this is the first study in medical education research that provides empirical evidence for the influence of different causal assumptions on study outcomes. It provides new insights into the plausible causal relationships in the emerging field of role modeling research.

Traditionally, researchers may test different models reflecting different causal assumptions, but only present the models with the highest explained variance or best statistical fit. The approach is reasonable if researchers are interested in the amount of variance explained by the set of variables in the models. However, for those interested in estimates of the causal effects of an exposure such as teaching performance on an outcome, selecting the ‘best’ model based on the best statistical fit or the greatest explained variance can be misleading. Statistical models with worse fit might yield estimates consistent for target causal effects while statistical models with ‘best’ fit might yield biased estimates. [22], [23] Because there was no agreement on the causal structure(s) relating teaching performance to role model types as of yet, this study did not aim to provide any additional theoretical support for one causal model over others. Rather, we aimed to enhance insights into the different plausible causal relations and their impact under different assumptions of the (unknown) data generating mechanism. To our knowledge, this is also the first study that applied modern DAGs theory in a medical education study [4], [6].

The differences in associations between the explored models could imply different interpretations for practice. For example, when considering models with the physician role model type as the outcome, the difference between the model without any mediating variables (model RM-phy1) and the model that included the role model type of person as a mediating variable (model RM-phy3) was large (OR (95% CI): 15.5 (12.3–19.5) versus 6.61 (5.14–8.51)). Results from these models may lead to different policy strategies for faculty who want to enhance their role modeling. Faculty who believe model RM-Phy1 is the best supported model (that is, only teaching performance is directly related to the physician role model type) would choose to invest in improving their teaching performance which would subsequently enhance their role modeling as physicians. However, faculty who believe RM-Phy3 is the best supported model – meaning that both teaching performance and role modeling as a person cause residents to perceive them as a role model physician - would be inclined to invest in qualities to enhance their role modeling as person, in addition to their investments in teaching performance.

In this study, only differences in the magnitudes (not directions) of the associations (or effects, if no uncontrolled confounding) were seen under the different assumed causal structures. The large effect sizes of the associations in this study resulted in large (and due to the big sample size, precise) odds ratios. In studies where the effect sizes or sample sizes are smaller, differences in assumed causal structures are more likely to affect magnitude, direction, and precision of estimates. For instance, differences in precision, that is, how narrow the confidence intervals are, will become important if imprecise and so-called statistically (in) significant results are included or excluded from subsequent considerations.

This study provides an overview of the plausible causal relationships of the effects of faculty’s teaching performance on their role modeling. This work can guide future empirical research in searching for evidence regarding specific causal relations in role modeling research. Future directions for research on the causal effects of teaching performance on role modeling include the exploration and identification of heterogeneity (including *interaction* and *modification*) of these effects by faculty’s background characteristics. These issues require stronger identification conditions and careful interpretation, which are beyond the scope of this paper.

Systematically drawing all plausible relationships between variables using DAGs can be a helpful first step to move the debate forward in areas where evidence supporting pre-specified causal assumptions is scarce. When researchers conclude that several causal models remain plausible, it can be valuable to report all those alternative models, so that readers may decide which assumptions they deem most plausible for their specific setting and which results they will value most from a study. When there are only a few plausible alternative models, it is often possible to report results from all these models concisely in one study. Results from alternative models are already reported in many studies, although this is mostly limited to univariable models versus multivariable adjusted models, that is models adjusted for appropriate confounding variables. [24] Likewise, alternative models examining mediation variables or heterogeneity could be added. It may not be feasible to explore and report the influence of all plausible assumptions for every study, as study reports will become unwieldy especially for an audience concerned with policy and practice. However, in studies where researchers do not report alternative models, they should at least be explicit about their causal assumptions and the potential influence of those assumptions on their study outcomes. Authors who do not have the opportunity to report the outcomes of alternative models in their main article could report the results of such models in an (online) appendix. With the advances in digitalization of the scientific literature, we hope and suspect that the opportunity to submit additional research findings in online appendices will be available in and even be encouraged by most scientific journals.

## Supporting Information

Appendix S1 **Results of the psychometric analyses.**
(DOC)
