# Supplementary material for: The Teacher, the Physician and the Person: Exploring Causal Connections between Teaching Performance and Role Model Types Using Directed Acyclic Graphs
Source: PLoS One. 2013 Jul 23;8(7):e69449. doi: 10.1371/journal.pone.0069449 (PMC3720648; doi:10.1371/journal.pone.0069449)
Supplement: Appendix S1 — Results of the psychometric analyses. (DOC) [file pone.0069449.s001.doc]

**Appendix S1**: Overview of the items and scales of the SETQ instrument, showing factor loadings and Cronbach’s alphas for internal consistency

| Item number | SETQ scale and items for measuring faculty’s teaching performance | Factor loading on primary scale | Cronbach’s alpha (α) |
| --- | --- | --- | --- |
|  | *Learning climate* |  | 0.89 |
| L1 | Encourages residents to participate actively in discussions | 0.75 |  |
| L2 | Stimulates residents to bring up problems | 0.75 |  |
| L3 | Motivates residents to study further | 0.73 |  |
| L4 | Stimulates residents to keep up with the literature | 0.68 |  |
| L5 | Prepares well for teaching presentations and talks | 0.56 |  |
|  | *Professional attitude towards residents* |  | 0.89 |
| P1 | Listens attentively to residents | 0.71 |  |
| P2 | Is respectful towards residents | 0.78 |  |
| P3 | Is easily approachable during on-calls | 0.85 |  |
| P4 | Is easily approachable for consultation (on the outpatients) | 0.83 |  |
|  | *Communication of goals* |  | 0.89 |
| C1 | States learning goals clearly | 0.80 |  |
| C2 | States relevant goals | 0.81 |  |
| C3 | Offers to conduct a mini-CEX (clinical examination exercise) regularly | 0.80 |  |
| C4 | Repeats stated learning goals periodically | 0.56 |  |
|  | *Evaluation of residents’ knowledge and skills* |  | 0.92 |
| E1 | Evaluates residents’ specialty knowledge regularly | 0.78 |  |
| E2 | Evaluates residents’ analytical abilities regularly | 0.76 |  |
| E3 | Evaluates residents’ application of knowledge to specific patients regularly | 0.80 |  |
| E4 | Evaluates residents’ medical skills regularly | 0.69 |  |
|  | *Feedback* |  | 0.92 |
| F1 | Regularly gives positive feedback to residents | 0.62 |  |
| F2 | Gives corrective feedback to residents | 0.79 |  |
| F3 | Explains why residents are incorrect | 0.83 |  |
| F4 | Offers suggestions for improvement | 0.80 |  |

The items shared the same subject “During my residency in [specialty], my attending faculty generally…”
